# A novel contralateral ulnar nerve transfer model for selective muscle reinnervation in upper motor neuron syndrome

**DOI:** 10.4103/NRR.NRR-D-24-00915

**Published:** 2025-09-03

**Authors:** Olga Politikou, Silvia Muceli, Leopold Harnoncourt, Florian Jaklin, Vlad Tereshenko, Udo Maierhofer, Matthias Luft, Christopher Festin, Gregor Laengle, Johanna Klepetko, Laurenz Pflaum, Konstantin D. Bergmeister, Oskar C. Aszmann

**Affiliations:** 1 Clinical Laboratory for Bionic Extremity Reconstruction, Department of Plastic, Reconstructive and Aesthetic Surgery, Medical University of Vienna, Vienna, Austria; 2 Department of Plastic Surgery and Hand Surgery, University Hospital Zurich, Zurich, Switzerland; 3 Department of Electrical Engineering, Chalmers University of Technology, Gothenburg, Sweden; 4 Division of Plastic and Reconstructive Surgery, Massachusetts General Hospital, Harvard Medical School, Boston, MA, USA; 5 Department of Plastic, Aesthetic and Reconstructive Surgery, Hospital of the Brothers of St. John of Gods, Paracelsus Medical University, Salzburg, Austria; 6 Department of Plastic, Reconstructive and Aesthetic Surgery, Medical University of Vienna, Vienna, Austria; 7 Department of Plastic, Aesthetic and Reconstructive Surgery, University Hospital St. Poelten, Karl Landsteiner University of Health Sciences, Krems, Austria

**Keywords:** electrophysiology, hemiplegia, muscle fiber type, muscle reinnervation, nerve regeneration, nerve transfer, spasticity, stroke, ulnar nerve, upper motor neuron lesion

## Abstract

Stroke and traumatic brain injury lead to upper motor neuron syndrome, which is characterized by muscle spasticity or paresis of varying severity depending on the lesion’s location and extent. Current treatments are mostly symptomatic with limited efficacy and significant side effects. Nerve transfer techniques, such as the contralateral L4 ventral root transfer in animal models and C7 root transfer in both animal and clinical studies, have been shown to reduce spasticity and improve function in upper motor neuron syndrome; however, they lack selectivity. Our hypothesis is that using a selective peripheral donor nerve from the contralateral side, rather than the entire nerve root, may represent an effective nerve transfer and provide a robust basis for future research on selective muscle reinnervation in upper motor neuron syndrome. Ten rats underwent a contralateral ulnar-to-ulnar nerve transfer procedure. Electrophysiological measurements were conducted twelve weeks post-surgery to assess successful reinnervation of the contralateral flexor carpi ulnaris muscle. Additionally, muscle biopsies of the reinnervated flexor carpi ulnaris were harvested to examine the muscle fiber type composition, cross-sectional area, and collagen content as well as compare them to naive counterparts. Axon quantification of the reinnervated nerves was also performed. All rats recovered uneventfully, maintaining the use of both paws post-surgery. Electrophysiological tests confirmed the successful reinnervation of the flexor carpi ulnaris muscle. Muscle fiber type composition, cross-sectional area, and collagen content did not show statistically significant changes. Axon counts indicated successful nerve regeneration without architectural disruption. In conclusion, we were able to demonstrate this novel contralateral nerve transfer model’s feasibility, reproducibility, and safety as well as achieve effective muscle reinnervation. This model provides a valuable tool for further research on selective muscle reinnervation and treatment of upper motor neuron syndrome, with potential implications for improving clinical outcomes in stroke and traumatic brain injury patients.

## Graphical abstract

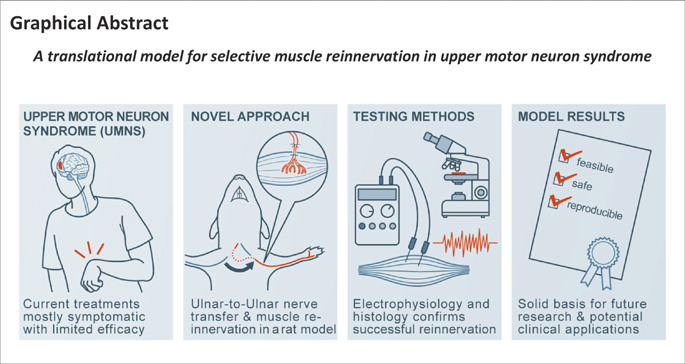

## Introduction

Stroke and traumatic brain injury are leading causes of upper motor neuron syndrome (UMNS) with broad clinical manifestations of positive and negative symptoms (Wissel et al., 2013; Angulo-Parker and Adkinson, 2018). One of the main positive features is muscle spasticity, which varies in severity depending on the location and extent of the lesions (Ward, 2012). The prevalence of disabling post-stroke spasticity ranges from 2% to 13% (Wissel et al., 2013), imposing substantial challenges regarding motor function rehabilitation and significant economic burdens on public health systems (Lundström et al., 2010). Despite these facts, current spasticity treatment is primarily symptomatic, with short-lasting effects and non-negligible side effects (Gresits et al., 2025). In preclinical models utilizing rats with upper motor neuron lesions, significant reduction of hindlimb spasticity was achieved through a contralateral L4 ventral root transfer (Zong et al., 2016). In recent clinical studies, the C7 root of the healthy side was transferred contralaterally to reinnervate the C7 root of the spastic hemiplegic side, resulting in significant improvement of functional scores and spasticity scale outcomes in patients with UMNS (Zheng et al., 2018; Xu, 2025). However, this approach remains non-specific for a condition with a diverse clinical presentation. Some muscles exhibit spasticity or paresis while others remain unaffected, thus requiring a more precise surgical plan. One such approach could involve selective distal nerve transfer surgery by rerouting healthy expendable nerves from unaffected muscles to the spastic muscles. This may simultaneously break the vicious cycle of spasticity and provide affected muscles with new cognitive control. Careful selection of suitable donors, which are still functional and under volitional control on the affected side, may be achievable in humans through thorough clinical examination; however, performing such an assessment in a rat model is not possible. Thus, utilizing a peripheral nerve from the contralateral intact side is necessary.

We developed a novel contralateral peripheral nerve transfer rat model and assessed its feasibility, morbidity, and effectiveness in selectively reinnervating muscles of the affected side. Our objective was to develop a robust and reproducible model, intended for future research on the effects of selective muscle reinnervation in spastic or paretic muscles in UMNS conditions.

## Methods

### Experimental design

Eight male Sprague–Dawley rat cadavers (Charles River Laboratories, Sulzfeld, Germany) were dissected to design the contralateral nerve transfer (CNT) procedure. Pre-spinal, pre-tracheal, and subcutaneous pathways were examined to determine the least invasive route with the lowest morbidity for contralateral passage. Among potential donor options, the ulnar nerve was selected due to its lower morbidity as finger and thumb flexor innervation is preserved. We considered the ulnar and median nerve at the level of their branching from the contralateral medial cord as suitable recipients and opted for the ulnar nerve due to the better size match and reduced morbidity until reinnervation occurs. The submuscular pre-tracheal route was chosen due to its short distance, enabling tension-free nerve coaptation and ensuring compression-free positioning of the donor nerve.

Ten male Sprague–Dawley rats (Charles River Laboratories) aged 8–10 weeks, weighing 280–320 g at the time of surgery, underwent ulnar-to-ulnar CNT performed by the same surgeon. Only male rats were used to minimize potential variability introduced by the estrous cycle in females, which can affect hormonal levels, muscle physiology, and behavioral outcomes (Beery and Zucker, 2011). The rats were housed under specific-pathogen-free (SPF) conditions. Environmental parameters were maintained at a constant temperature of 22°C, humidity of approximately 60%, and a 12-hour light/dark cycle. Animals were kept in pairs (2 per cage) with ad libitum access to food and water.

For each procedure, anaesthesia was induced with intraperitoneal ketamine (100 mg/kg, Ketamidor, VetViva Richter, Wels, Austria) and intraperitoneal xylazine (5 mg/kg, Rompun, Elanco, Monheim am Rhein, Germany) and maintained by volume-controlled ventilation (40% O_2_, room air, 1.5%–2% isoflurane, IsoFlo Zoetis Parsippany-Troy Hills Township, NJ, USA) following orotracheal intubation. Piritramide (0.3 mg/kg, Hameln Pharma, Hameln, Germany) was administered subcutaneously for analgesia. Postoperatively, the drinking water was mixed with piritramide and glucose (30 mg piritramide and 30 mL 10% glucose dissolved in 250 mL drinking water) and administered ad libitum for pain relief during the first 7 postoperative days. All animals were examined daily by an animal keeper for pain, weight loss, impairments in daily activities, wound dehiscence, and infection. Twelve weeks after surgery, electrophysiological measurements of the reinnervated flexor carpi ulnaris (FCU) muscle were performed and nerve and muscle biopsies were obtained for histomorphological examination. Four naive Sprague–Dawley rats aged 8–10 weeks were used as external controls. After the experimental tests, animals were euthanized with a lethal dose of pentobarbital (Release 300 mg/kg, WDT, Garbsen, Germany) injected intracardially under deep anaesthesia.

Experiments were planned, conducted, and reported in adherence to the ARRIVE (Animal Research: Reporting of *In Vivo* Experiments) guidelines (Percie du Sert et al., 2020). Ethical approval was obtained from the Ethics Committee of the Medical University of Vienna and the Austrian Ministry for Research and Science (reference number BMBWF – 2020-0.468.730, approval date August 17, 2020). Additionally, the experiments strictly followed the principles of laboratory animal care outlined by the Federation of European Laboratory Animal Science Associations (FELASA).

### Contralateral peripheral nerve transfer model

**Figure 1:**
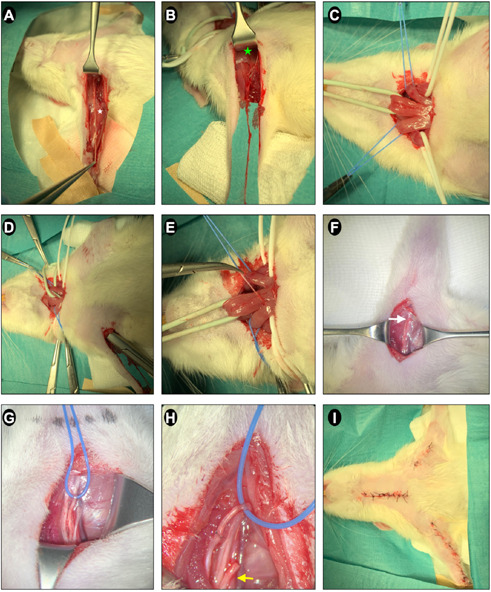
Surgical steps for the contralateral ulnar to ulnar nerve transfer. (A) Through a long incision from the paw to the upper arm, the motor branch of the ulnar nerve is dissected from the loge de Guyon up to its origin from the medial cord. White asterisk: flexor carpi ulnaris muscle. (B) The pectoralis major muscle (green asterisk) has to be divided for 2–3 mm to expose the medial cord. (C) Through a second midline cervical incision, the cervical muscles are bluntly dissected and marked with vessel loops to facilitate traction. Green vessel loop: sternohyoid muscle, blue vessel loops: sternomastoid muscle, white vessel loops: cleidotrapezius muscle. (D) A retroclavicular tunnel passing through the scalenus triangle is created using thin Metzenbaum scissors under direct view. (E) A micro-needle holder is passed from the cervical to the arm incision and the ulnar nerve is gently pulled to the midline until it reaches its maximal length. The nerve is then placed underneath the muscles for a shorter path (not depicted). (F) Through a third incision on the proximal contralateral upper arm, the pectoralis major muscle is divided for 2–3 mm to expose the medial cord. Then, the donor ulnar nerve (white arrow) is brought from the midline to the upper arm using the same procedure. (G) The medial cord is dissected and the recipient ulnar nerve is marked with a vessel loop (blue). (H) The recipient ulnar nerve is transected 2 mm distal to its branching from the medial cord and a microsurgical epineural coaptation using 2 Nylon 11-0 single interrupted sutures is performed. Yellow arrow: the proximal stump of the recipient ulnar nerve. (I) Overview of the three approaches following skin closure.

The rat was positioned supine with its paws fixed in full supination. Preoperative preparation included shaving and disinfecting the surgical field. A volar longitudinal skin incision was made from Guyon’s canal to the proximal third of the upper arm. Under microscopic magnification, the ulnar nerve’s trifurcation into deep and superficial branches was identified in Guyon’s canal and the motor branch was dissected using microscissors, ensuring protection of the ulnar artery. The ulnar nerve, situated beneath the FCU muscle, was carefully dissected up to the elbow without excising the muscle. Intramuscular dissection was necessary near the muscle’s origin at the medial epicondyle due to the nerve’s path between the FCU muscle heads and its partial attachment to the fascia. Care was taken to avoid injury to the recurrent branch of the ulnar artery. In the upper arm, the ulnar nerve, located medial to the brachialis muscle, was easily dissected. The pectoralis major muscle was longitudinally incised for approximately 2–3 mm, allowing cranial retraction with a Langenbeck retractor to trace the nerve underneath. The nerve’s origin from the medial cord marked the end of the proximal dissection, achieving a maximal donor nerve length of 5.5 cm (**Figure 1A** and **B**).

A 2 cm incision was made along the midline of the ventral cervical area. Submandibular glands were protected and retracted cranially. The bellies of the sternohyoid, sternomastoid, and cleidotrapezius muscles were bluntly separated and marked with vessel loops (**Figure 1C**). Subsequently, a tunnel was created from the arm incision to the midline incision using thin Metzenbaum scissors (Fine Science Tools GmbH, Heidelberg, Germany, product number 03-502/18) with direct visualization of the brachial plexus, passing beneath the pectoralis major muscle and through the scalene triangle. Care was taken to avoid major vessel injury. A micro-needle holder (product number B-18-8.2, S&T Microsurgical Instruments, Birmingham, AL, USA) was passed from the cervical incision through the tunnel toward the arm incision. The ulnar nerve was carefully grasped and pulled through the tunnel until reaching its maximum length and temporarily placed into the midline wound (**Figure 1D** and **E**). This step is shown in **Additional Video 1**.

**Figure 2:**
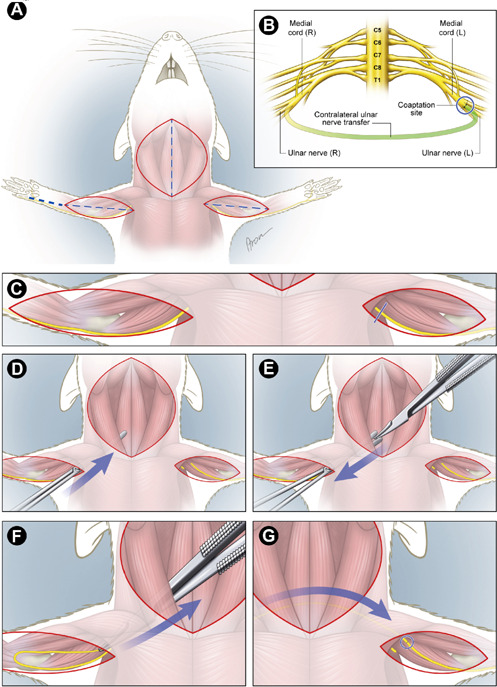
Schematic representation of the surgical steps. (A) Skin incisions. (B) Illustration of the brachial plexus of both sides and depiction of the principle of the contralateral ulnar-to-ulnar nerve transfer. (C) The donor ulnar nerve has to be freed from the sulcus in the elbow. (D) The submuscular trajectory of the created tunnel from the upper arm to the cervical incision. (E) The retrograde passage of the micro-needle holder through the tunnel. Care needs to be taken to avoid an injury to the subclavian vessels. (F) The donor ulnar nerve is gently grabbed and pulled from the upper arm to the midline. (G) The donor nerve is then passed to the contralateral upper arm using the same method. This is followed by epineural coaptation with the recipient ulnar nerve.

A third incision measuring approximately 1 cm was made on the proximal/middle third of the left upper arm to expose the cords and branches of the brachial plexus. Under microscopic magnification, the ulnar nerve was dissected at its origin from the medial cord and transected as proximally as possible leaving a 2–3 mm stump. The donor ulnar nerve was passed in a submuscular plane underneath the sternohyoid, sternomastoid, and cleidotrapezius muscles from the midline to the left upper arm incision with the same procedure (**Figure 1F** and **G**). Lastly, a tension-free epineural coaptation was performed between the donor and recipient ulnar nerve using two Nylon 11/0 single interrupted sutures (**Figure 1H**). The wounds were irrigated with saline and closed using braided resorbable 5/0 sutures (**Figure 1I**). **Figure 2** depicts a schematic representation of the whole procedure.

### Electrophysiological assessment

For the electrophysiological examination of the reinnervated FCU muscle, rats were anesthetized with intramuscularly administered ketamin (0.05 mL/100 g, Ketamidor) and dexmedetomidin (0.01 mL/100 g, Dexdomitor, Ismaning, Germany). Half of the dose was administered in a 30-minute interval following control of the corneal and toe pinch reflexes. Anesthesia with isoflurane was avoided due to its effects on reflex suppression in the spinal cord (Jinks et al., 2002).

We recorded the electrical response of the FCU muscle when the transferred ulnar nerve was stimulated electrically. For this purpose, two metal pins were used for nerve stimulation and bipolar fine wires (diameter: 0.2 mm; A-M Systems, Carlsborg, WA, USA) for muscle recordings. The ulnar nerve was exposed in the upper arm and the stimulation electrodes were placed 5 mm proximal to the medial epicondyle. The fine wires were then placed distal to the motor nerve entry point.

Monopolar pulses with a pulse width of 100 μs, a frequency of 10 Hz, and amplitude that elicited supramaximal contraction were delivered 5 times (DS3, Digitimer Ltd., Hertfordshire, UK). The corresponding muscle signals were acquired with a bioelectrical amplifier (Quattrocento, OT Bioelettronica, Torino, Italy) with a gain of 150, a 16-bit resolution and a 10240 Hz sampling frequency.

### Changes in muscle fiber type composition

Muscle fiber type composition was assessed using immunofluorescence staining with antibodies specific to myosin heavy chain isoforms. After the electrophysiological assessment, the entire reinnervated FCU muscle of three animals and both FCU muscles of four naive animals were carefully dissected and extracted. Using a 10 mm blade, the tendinous part was excised at the musculotendinous junction and the muscle was axially halved to obtain sections from its middle part. The samples were embedded in optimal cutting temperature compound using liquid-nitrogen-cooled isopentane and stored at –80°C for 24 hours. Subsequently, 10 μm-thick cross sections of the embedded muscles were sectioned. A previously described immunofluorescence protocol was applied (Luft et al., 2021). Initially, wheat germ agglutinin conjugated Alexa Fluor 594 (1:250, Alexa Fluor^TM^ 594, WGA-AF594, Cat# W11262, RRID: AB_2288806) was applied at room temperature for 10 minutes to stain the muscle fiber membrane (Thermo Fisher Scientific, Waltham, MA, USA). Primary antibodies against myosin heavy chain (MHC)-I (1:50, mouse monoclonal IgG2b, anti-slow skeletal myosin heavy chain, Cat# BA-F8, RRID: AB_2235587), MHC-IIa (1:600, mouse monoclonal IgG1, anti-fast skeletal myosin heavy chain type IIa, Cat# SC-71, RRID: AB_2147165), and MHC-IIb (1:100, mouse monoclonal IgM, anti-fast skeletal myosin heavy chain type IIb, Cat# BF-F3, RRID: AB_2266724) diluted in 0.1 M phosphate-buffered saline (PBS) with 10% goat serum were applied at room temperature for 60 minutes (Developmental Studies Hybridoma Bank, Iowa City, IA, USA). Subsequently, secondary antibodies against Alexa Fluor 633 IgG2b (1:250, Alexa Fluor^TM^ 633 goat anti-mouse IgG2b, Cat# A-21146, RRID: AB_2535782), Alexa Fluor 488 IgG1 (1:250, Alexa Fluor^TM^ 488 goat anti-mouse IgG1, Cat# W11262, RRID: AB_2534114), and Alexa Fluor 555 IgM (1:250, Alexa Fluor^TM^ 555 goat anti-mouse IgM, Cat# A-21426, RRID: AB_2535857) were applied at room temperature for 60 minutes (Thermo Fisher Scientific). Digital images of the whole muscle cross sections were acquired using a whole-slide scanner (Vectra® Polaris^TM^, Akoya Biosciences, Inc., Malborough, MA, USA) at 20× magnification. Images were analyzed using the Halo imaging analysis platform version 3.2.1851.421 (Indica Labs, Inc., Albuquerque, NM, USA) by a single independent investigator using a previously published protocol (Tereshenko et al., 2022). The proportions of type I (yellow), type IIa (green), type IIb (red), and type IIx (unstained) fibers were calculated based on fluorescence labeling.

### Cross-sectional area and collagen content of the reinnervated muscles

Collagen content and muscle cross-sectional area were quantified from Picrosirius Red-stained sections using digital image analysis. Four reinnervated FCU muscles and eight naive FCU muscles were harvested. As described before, the tendinous part was removed, and the muscle was axially halved to obtain samples from its middle part. Muscles were immersed and fixed in freshly prepared 4% paraformaldehyde in 0.1 M PBS for a maximum of 24 hours followed by a 24-hour rinse with PBS. Subsequently, specimens were immersed in ethanol solutions of increasing concentration, followed by xylene clearing, wax infiltration, and paraffin-embedding. Semithin cross-sections of 4 μm were deparaffinized, rehydrated, and incubated with Picro Sirius red staining (Picro Sirius Red Staining Kit, ab150681, Abcam, Cambridge, MA, USA) for 60 minutes and then mounted with Entellan (1.07961, Sigma-Aldrich, St. Louis, MO, USA). Slide scanning was performed using a whole-slide scanner (Vectra® Polaris^TM^, Akoya Biosciences, Inc.) at 20× magnification. The muscle cross-sectional area (μm^2^), collagen-positive tissue area (μm^2^), and the percentage of total collagen area to total tissue area (%) were calculated using the Halo imaging analysis platform version 3.2.1851.421 (Indica Labs, Inc.). Collagen was segmented based on its red birefringence signal, and the percentage of collagen relative to the total muscle area was calculated. Total cross-sectional area was determined by outlining the entire muscle contour.

### Axon quantification of the recipient nerve

Following inspection for continuity at the nerve coaptation site, two 2 mm nerve biopsies were taken proximal and distal to the coaptation and fixed in 2.5% glutaraldehyde for 24 hours followed by a 24-hour rinse with 0.1 M cacodylate buffer. After a 2-hour pre-fixation with 2% osmium tetroxide (Sigma-Aldrich), specimens were dehydrated in ethanol solutions of increasing concentration and embedded in epon resin. Subsequently, 1 μm semithin cross-sections were obtained using a Leica Ultracut UCT (Leica Microsystems, Wetzlar, Hesse, Germany), stained for 60 seconds with toluidine blue (Sigma-Aldrich), and mounted with gelatin. Whole slide scanning was performed with Vectra® Polaris^TM^ (Akoya Biosciences, Inc.) at 40× magnification. The total numbers of myelinated axons in the proximal and distal segments were counted using a semi-automated axon count protocol with Fiji software (Fiji Is Just ImageJ, open-source platform, Max Planck Institute of Molecular Cell Biology and Genetics, Dresden, Germany), as previously described (Engelmann et al., 2020).

### Statistical analysis

Statistical analysis was conducted with the SPSS 29.0.0.0 software (IBM, Armonk, NY, USA). Data were assessed for normal distribution and expressed as mean and standard deviation (SD) values of each group. The average of two extracted FCU muscles from the same naive animal was calculated and used for comparison for the operated FCU muscles. The muscle cross-sectional area, collagen content, and muscle fiber type composition (%) were compared using an unpaired two-sided *t*-test. The *P*-value of < 0.05 was considered statistically significant.

## Results

### Contralateral nerve transfer surgery and behavioral evaluation

To assess the feasibility and safety of our contralateral ulnar-to-ulnar nerve transfer model, we first evaluated surgical outcomes and postoperative behavior. Ιn total, a ulnar-to-ulnar CNT was performed in ten adult rats with a mean weight of 441 ± 61 g. The mean surgery time was 143 ± 28 minutes. All animals had an uneventful recovery and were able to use both paws immediately after surgery (**Additional Videos 2** and **3**). No complications regarding wound healing or infection were observed and the animals did not show any signs of pain or distress. These results confirm the reproducibility and minimal morbidity of the surgical procedure.

### Electrophysiological assessment

**Figure 3:**
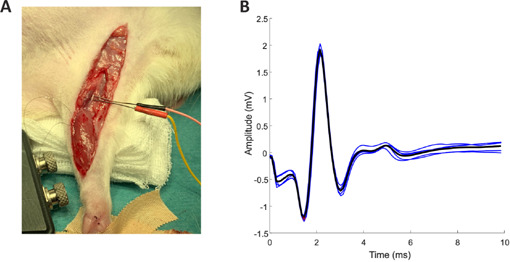
Electrophysiological confirmation of muscle reinnervation after contralateral nerve transfer. (A) The electromyography setup. Note the exposed ulnar nerve with selective bipolar stimulation and the selective bipolar recording from the flexor carpi ulnaris muscle. (B) An M-wave is elicited from the flexor carpi ulnaris muscle of the recipient side following bipolar stimulation of the exposed ulnar nerve in the upper arm, indicating successful reinnervation of the muscle (blue: M-wave from single trials; black: waveform average).

To confirm successful reinnervation of the FCU muscle, electrophysiological measurements were performed 12 weeks postoperatively. It was possible to elicit an M-wave in all animals. The setup for the recording and a representative example of the muscle’s electrical response are shown in **Figure 3A** and **B**, respectively. The mean M-wave amplitude recorded at stimulation eliciting supramaximal contraction was 3.99 ± 2.47 mV with a mean latency of 0.98 ± 0.16 ms. These findings demonstrate that the transferred contralateral ulnar nerve achieved functional muscle reinnervation.

### Changes in muscle fiber type

**Figure 4:**
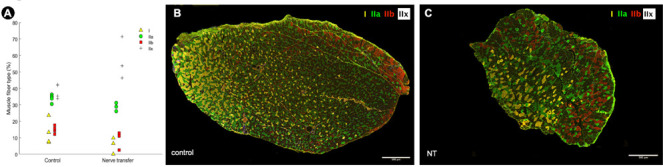
Muscle fiber type composition after selective reinnervation of the flexor carpi ulnaris muscle. (A) Diagram of the percentage (%) of muscle fiber types in the control and NT groups. (B) Whole cross sections of the flexor carpi ulnaris muscle following immunofluorescence staining for muscle fiber type composition in the control group and (C) in the NT group. Note the smaller size of the reinnervated muscle compared to the control muscle, while maintaining a similar muscle fiber type composition. Magnification 20×, scale bars: 500 μm. Type I: yellow, type IIa: green, type IIb: red, type IIx: unstained, DAPI: blue, membrane staining: orange. NT: Nerve transfer group.

We next investigated whether selective reinnervation altered the muscle fiber type composition of the FCU. The total number of FCU muscle fibers was reduced in the CNT group; however, it was not statistically significant (*P* = 0.052). The percentage of slow type I and fast type IIA and IIB muscle fibers decreased while the percentage of unstained fibers slightly increased in the CNT group compared to the naive group, but without a statistically significant effect (*P* = 0.184, *P* = 0.052, *P* = 0.176, *P* = 0.118, respectively). Results are presented in **Figure 4**. Thus, selective reinnervation preserved the overall muscle fiber type profile.

### Cross-sectional area and collagen content of the reinnervated flexor carpi ulnaris muscle

**Figure 5:**
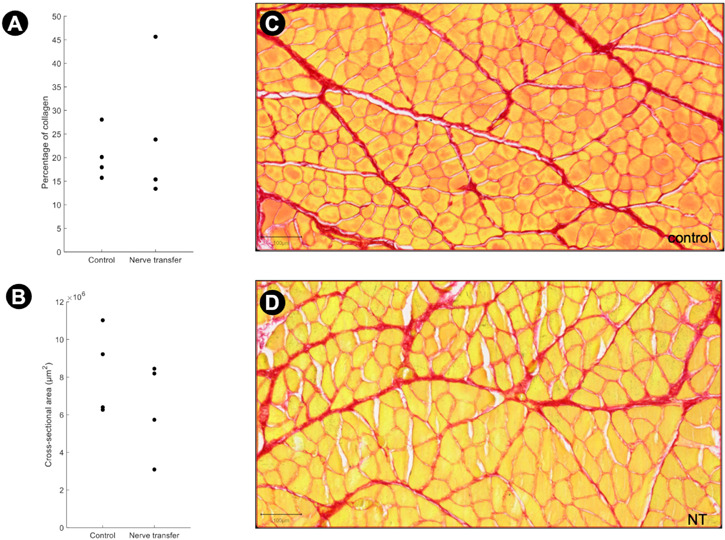
Muscle size and fibrosis assessment following contralateral nerve transfer. (A, B) Diagrams of the percentage of collagen in relation to total muscle area (A) and the total cross-sectional area (B) in the control and nerve transfer (NT) group. (C, D) Muscle staining with picrosirius red for collagen content quantification between the control (C) and NT groups (D). Magnification 10×, scale bars: 100 μm. Muscle fiber: orange, collagen: red.

We then assessed whether reinnervation influenced FCU muscle size and fibrosis. The mean muscle cross-sectional area was reduced in the CNT group with 6.400.000 ± 2.500.000 μm^2^ compared to 8.200.000 ± 2.300.000 μm^2^ in the naive group. This, however, was not statistically significant (*P* = 0.317). The total collagen-positive area was 1.600.000 ± 941.000 μm^2^ and 1.600.000 ± 486.000 μm^2^ while the percentage of collagen-positive area to total muscle area was 24.5 ± 14.8 and 20.5 ± 5.3 in the CNT and naive group, respectively. The results were not statistically significant (*P* = 0.921 and *P* = 0.631) and are presented in **Figure 5**. These results indicate that muscle reinnervation was not associated with significant atrophy or fibrosis.

### Myelinated axon count proximal and distal to the coaptation site

**Figure 6:**
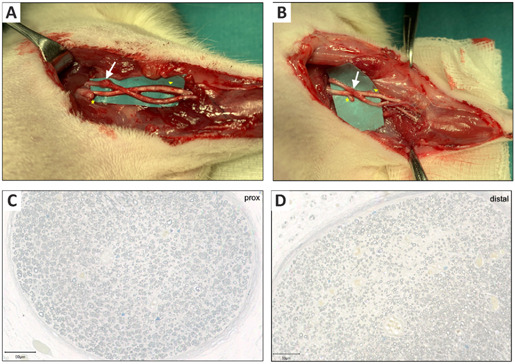
Axonal regeneration and structural integrity of the reinnervated ulnar nerve. (A, B) Macroscopic assessment of the coaptation site (white arrow) 12 weeks after the contralateral ulnar-to-ulnar nerve transfer in two different animals (animal 1: A, animal 2: B). No rupture and no aberrant reinnervation by the remaining proximal stump of the recipient nerve (yellow asterisk) were noted. The ulnar nerve is crossing over the median nerve (yellow triangle). (C, D) Toluidine blue staining following 2-hour pre-fixation with osmium tetroxide 2% and resin embedding of ulnar nerve biopsies proximal (C) and distal (D) to the coaptation site. Magnification 20×, scale bars: 50 μm. prox: Proximal.

To verify structural continuity and regeneration across the nerve coaptation, axon counts were performed proximally and distally. In all cases, we could demonstrate the nerve’s continuity and the absence of aberrant reinnervation through the proximal stump or other nerves (**Figure 6A** and **B**). The mean myelinated axon count of the recipient and donor was 6206 ± 1081 and 2907 ± 1118, respectively, with a mean donor/recipient ratio of 0.46. The intraneural architecture of the reinnervated ulnar nerve did not appear disturbed as shown in **Figure 6C** and **D**. These data confirm successful axonal regeneration across the coaptation site without structural disruption.

## Discussion

In this study, we present an innovative contralateral peripheral nerve transfer model designed for the selective reinnervation of a targeted muscle group on the affected side in UMNS. We evaluated the feasibility and reliability of this model and provided a comprehensive description of the surgical procedure. Additionally, we assessed and validated its efficacy using electrophysiological and histomorphometric analyses.

The concept of using contralateral roots as donors for ipsilateral reinnervation is not new. The initial report by Chen and Gu (1994) described the transfer of the contralateral C7 cervical root (CC7) for limb reanimation in a rat model. Since then, various CC7 rat models have been developed for the treatment of severe brachial plexus injuries, with or without the use of a nerve graft and also targeting different nerve recipients (Wang et al., 2013; Jiang et al., 2018). This concept has also been clinically applied for obstetrical and post-traumatic brachial plexus injuries in humans with numerous reports indicating favorable outcomes (Liu et al., 2020; Chen et al., 2023).

Recently, the contralateral transfer of cervical, lumbar, or sacral roots has been utilized to reduce spasticity in the upper and lower extremities in cases of spastic hemiplegia, yielding promising results (Zong et al., 2016; Zheng et al., 2018; Qiu et al., 2019; Feng et al., 2022; Luo et al., 2023; Pan et al., 2023; Zhu et al., 2024; Yang et al., 2024). In our animal model, we used a more selective donor from the contralateral side rather than the entire root. Moreover, we selectively reinnervated the ulnar nerve rather than an entire root or trunk. To date, there is only one animal study reporting the contralateral transfer of a peripheral nerve (radial nerve) to selectively reinnervate the medial cord of the brachial plexus (Bertelli et al., 1999). In our study, we utilized a direct contralateral ulnar-to-ulnar nerve transfer, performing an end-to-end coaptation without an intervening nerve graft. This design differs from the approach by Sinis et al. (2006), in which a contralateral median nerve was used as a donor and an autologous ulnar nerve graft was employed to facilitate reinnervation of the median nerve on the impaired side. In the Sinis model, the autologous nerve graft, rather than the donor nerve itself, was transferred contralaterally. This indirect approach achieved reinnervation via the contralateral median nerve but introduced an additional structural component—the graft—that may influence the specificity and efficiency of axonal regrowth. By omitting an intermediate graft in our model, we aimed to reduce potential points of axonal divergence and simplify the pathway for regenerating fibers. This direct coaptation approach may enhance reinnervation selectivity and allow for more precise functional recovery, given that axons are guided directly to the recipient nerve.

Overall, the need for selectivity in donor selection and recipient reinnervation is rising. Recent studies explored the anatomical feasibility of a more selective targeted reinnervation of the hemiplegic side with the CC7 targeting one to two roots with more specific functional patterns (Zhao et al., 2022; Zhu et al., 2024; Yan et al., 2024) or through distal nerve transfers (Jaloux et al., 2022; Waxweiler et al., 2022). In humans, the effects of treating spastic or paretic muscles in UMNS through selective muscle reinnervation with distal nerve transfers, as opposed to non-specific root transfers, have not yet been investigated clinically, electrophysiologically, or histomorphometrically. Our aim was to establish an animal model to treat UMNS manifestations such as spasticity and paresis on the affected side using healthy donors from the contralateral side. The mechanism by which our contralateral ulnar-to-ulnar nerve transfer model facilitates muscle reinnervation and addresses UMNS manifestations lies in the targeted reinnervation of affected muscles, bypassing disrupted upper motor pathways. In UMNS, spasticity and paresis arise from disrupted descending motor control due to cortical or subcortical lesions (Ward, 2012; Wissel et al., 2013). Traditional treatments often fail to restore voluntary control or reduce spasticity effectively because they do not directly address the impaired neuromuscular connections. In our model, by rerouting a functional nerve from the unaffected contralateral side, we provide a new source of motor input to the affected muscles, bypassing the central lesion. The contralateral ulnar nerve is selectively connected to the denervated ulnar nerve on the affected side, which allows axonal regeneration to occur along a direct path to the target muscle. This reinnervation potentially restores voluntary control over the FCU, offering a novel approach to managing UMNS symptoms by restoring functional input without involving the impaired motor pathways. Clinically, this could translate to performing distal nerve transfers on the ipsilateral affected side, which remain under volitional control, to reinnervate spastic, non-volitionally controlled muscles.

In UMNS conditions such as traumatic brain injury, cerebral palsy, stroke, or spinal cord injury, not all muscles on the affected side are equally impacted (Urban et al., 2010). Patients often present with a mosaic of clinical involvement. In UMNS rat models, it is challenging to observe and quantify spasticity, and distinguishing between affected and unaffected muscles on the “hemiplegic” side can be unreliable. Therefore, we developed an animal model using a peripheral nerve from the contralateral side, which remains unaffected. The potential clinical application of this model would involve distal nerve transfer with a volitionally controlled donor nerve from the affected side, rather than the same contralateral transfer.

In designing our study, we selected the ulnar nerve as the donor and recipient, guided by the findings that ulnar nerve impairment alone does not result in significant limb function loss, in contrast to median nerve impairment (Tos et al., 2009). This choice allowed us to minimize morbidity in line with the 3Rs principle (Replacement, Reduction, Refinement), which emphasizes reducing harm to experimental animals. Consequently, we did not expect observable functional deficits and thus opted against functional testing. Instead, we relied on electrophysiological studies as a more objective method to confirm reinnervation. This approach differs from that of Sinis et al. (2006), who used the median nerve and a longer observational period, likely due to the greater functional impact associated with median nerve impairment. Moreover, we harvested the ulnar nerve from its most distal part and dissected it up to its branching from the medial cord. This marked the end of the dissection, permitting a maximal length of 5.5 cm, which is sufficient to reach the contralateral ulnar nerve for a tension-free coaptation. With this donor/recipient nerve selection, we achieved minimal morbidity on both the donor and recipient sides until reinnervation occurred. The paw remained fully functional through median nerve innervation, as demonstrated in our direct postoperative video. Additionally, the donor/recipient size match is optimal, and the myelinated axons ratio is approximately 0.5, which is sufficient for effective reinnervation.

Our study investigated the effects of selective muscle reinnervation on muscle fiber composition and collagen content in the flexor carpi ulnaris muscle and did not reveal any statistically significant differences compared to the naive muscles. Similarly, no alterations in the percentage of slow and fast muscle fibers as well as collagen content were observed in the reinnervated FCU muscles. Additionally, qualitative assessments revealed no architectural changes in the reinnervated muscle fibers. These findings suggest that cognitive muscle reinnervation, using a donor which innervates a target with the same or similar muscle fiber type composition, may not significantly impact muscle fiber composition. However, further studies with larger sample sizes are warranted to elucidate the long-term effects of cognitive muscle reinnervation on muscle structure, particularly in conditions such as spinal cord injury, traumatic brain injury, and stroke-induced hemiplegia.

Despite the promising results, several limitations of this study should be acknowledged. First, the lack of functional tests to assess limb performance after reinnervation may limit the evaluation of functional recovery in this model. We opted for electrophysiological measurements to confirm reinnervation due to the minimal functional impairment associated with ulnar nerve transection; however, this choice may restrict the assessment of fine motor improvements. Second, the observational period was limited to 12 weeks post-surgery. Although this duration was sufficient to confirm reinnervation, longer-term studies may be needed to evaluate the sustainability of functional recovery and the potential development of any delayed complications in the reinnervated muscle. Third, the use of a single donor-recipient nerve pair (ulnar-to-ulnar) restricts the generalizability of the model. Future studies may explore additional nerve pairs and further investigate the applicability of this model to other muscles affected by upper motor neuron syndrome. Finally, the study was conducted in a rat model, which, while valuable for preclinical research, may not fully replicate the anatomical and physiological complexities seen in human nerve transfer surgeries. Further research in larger animal models or clinical trials will be necessary to validate the translational potential of this approach.

In summary, we have established a robust and reproducible animal model for a contralateral peripheral nerve transfer, including detailed surgical instructions as well as efficacy and reliability assessments. This model holds significant potential for advancing research on selective reinnervation of spastic or paretic muscles in UMNS, paving the way for innovative treatments and improved outcomes in clinical settings.

## Additional files:

***Additional Video 1:*** *Ulnar nerve passage and positioning in the midline wound.*



***Additional Video 2:*** *Bilateral paw use following contralateral ulnar nerve transfer: early postoperative recovery.*



***Additional Video 3:*** *Functional recovery of grasping and feeding with both forelimbs.*



## Data Availability

*All relevant data are within the paper and its Additional files.*
